# Prenatal Glucose Intolerance and Child Neurodevelopmental Disorders

**DOI:** 10.1001/jamanetworkopen.2025.41657

**Published:** 2025-11-05

**Authors:** Luke P. Grosvenor, Erica P. Gunderson, Yinge Qian, Stacey Alexeeff, Jennifer L. Ames, Lauren A. Weiss, Elizabeth Sahagun, Paul Ashwood, Robert Yolken, Yeyi Zhu, Judy Van de Water, Lisa A. Croen

**Affiliations:** 1 Division of Research, Kaiser Permanente Northern California, Pleasanton; 2 Bernard J. Tyson Kaiser Permanente School of Medicine, Pasadena, California; 3 University of California at San Francisco; 4 University of California at Davis; 5 Johns Hopkins University, Baltimore, Maryland

## Abstract

**Importance:**

Gestational diabetes has been associated with risk of neurodevelopmental disorders (NDD). An improved understanding of this association can inform prevention strategies and elucidate underlying mechanisms.

**Objective:**

To determine associations between prenatal glucose intolerance and NDD and examine differences by gestational timing and child sex.

**Design, Setting, and Participants:**

This population-based case-control study examined data from electronic health records from mother-child pairs in an integrated health system in northern California. Children born January 1, 2011, to December 31, 2018, and their mothers were eligible; children were followed up for outcomes through 2023. Data were analyzed from February 2024 to March 2025.

**Exposures:**

Gestational diabetes was determined from routine prenatal test results and categorized as diagnosed early (less than 24 weeks), standard (24 to 28 weeks), or late (more than 28 weeks) in gestation. Prenatal subclinical impaired glucose tolerance (IGT) was defined by elevated glucose screening tests and neither GDM diagnosis nor treatment.

**Main Outcomes and Measures:**

Autism spectrum disorder (ASD) and developmental delay were determined from medical records. Adjusted odds ratios (aOR) for associations between prenatal exposures and NDD were estimated using multivariable logistic regression models, adjusted for child sex, birth year, maternal age, race and ethnicity, education, parity, gestational age at prenatal care entry, and prepregnancy body mass index. Effect modification was evaluated by GDM diagnosis timing and sex.

**Results:**

A total of 4546 mother-child pairs (median [IQR]) age of diagnosis: ASD, 3.0 [2.0-5.0] years; developmental delay, 2.0 [1.0-3.0] years; 2697 male children [59.3%]) were included in the study, of which 403 mothers (8.9%) had GDM and 64 (1.4%) had IGT; 683 children [15.0%] had ASD, 2054 [45.2%] had developmental delay, and 1809 [39.8%] were controls. GDM was not associated with increased odds of ASD (aOR, 1.15 [95% CI, 0.83-1.60]) or developmental delay (aOR, 1.24 [95% CI, 0.98-1.57]) overall. In sex-stratified analyses, GDM was associated with increased odds of ASD only among females (females: aOR, 2.05 [95% CI, 1.15-3.56]; males: aOR, 0.93 [95% CI, 0.62-1.37]; *P* for interaction = .04). When assessed by timing, early GDM was associated with increased odds of ASD among females (aOR, 3.23 [95% CI, 1.11-8.91]) but not among males (aOR, 0.78 [95% CI, 0.38-1.56]; *P* for interaction = .02). There were no associations between standard or late GDM and ASD in either sex. Prenatal IGT was associated with increased odds of developmental delay among females only (females: aOR, 3.25 [95% CI, 1.34-8.68]; males: aOR, 1.07 [95% CI, 0.50-2.39]; *P* for interaction = .08).

**Conclusions and Relevance:**

In this case-control study, GDM was associated with NDD in a gestational timing- and sex-specific manner. IGT associations with NDD were also sex-specific, adding to a body of research demonstrating influences of prenatal IGT on child outcomes.

## Key Points

**Question:**

Do associations between prenatal glucose intolerance and neurodevelopmental disorders, including autism spectrum disorder and developmental delay, differ by gestational timing of exposure and child sex?

**Findings:**

In this case-control study of 4546 mother-child pairs, gestational diabetes diagnosed earlier in pregnancy was associated with increased odds of autism spectrum disorderamong female children only. Prenatal exposure to subclinical impaired glucose tolerance was associated with increased odds of developmental delay, also among females only.

**Meaning:**

These findings suggest that evidence for sex differences in the associations of both gestational diabetes and prenatal impaired glucose tolerance with risk of child neurodevelopmental disorders.

## Teaser

This case-control study examined associations between prenatal glucose intolerance and neurodevelopmental disorders, such as autism spectrum disorder and developmental delay, and differences by gestational timing and child sex.

## Introduction

Cardiometabolic conditions of pregnancy, including gestational diabetes, obesity, and preeclampsia, have been associated with increased risk of autism spectrum disorder (ASD).^1,2,3,4,5^ Prevalence of GDM has risen considerably since 2000 in the US and globally^6,7^ and disparities exist across race and ethnicity groups and country of origin,^8,9,10^ underscoring the importance of characterizing associations with child health outcomes. Standardized screening, diagnostic, and treatment practices for gestational diabetes represent potential avenues for targeted prevention of adverse child outcomes. Improved understanding of associations between prenatal glucose intolerance and neurodevelopmental disorders (NDD), which are increasingly common,^11,12^ can also provide insight into etiologic mechanisms.

Gestational diabetes can impact fetal neurodevelopment through multiple mechanisms, including oxidative stress,^13^ epigenetic modifications,^14^ or systemic inflammation,^15^ which cause dysregulation of neuronal migration and impaired brain connectivity.^16^ Pregnant individuals with elevated glucose screening levels who do not receive a gestational diabetes diagnosis are likely still impacted by impaired glucose tolerance (IGT), a subclinical form of hyperglycemia.^17^ Despite being a subclinical indicator of mild glucose intolerance, IGT has been associated with maternal inflammatory markers similar to gestational diabetes^18^ and merits evaluation as a risk factor for NDD.

Gestational diabetes may impact fetal brain development differently depending on gestational timing of exposure. Using diagnosis date as a proxy for disorder onset, 2 studies^1,19^ reported increased risk of ASD associated with gestational diabetes diagnosed before 26-weeks’ gestation but not later in pregnancy. Another^2^ reported greater risk associated with gestational diabetes diagnosis between 27 to 30 weeks, compared with earlier or later in gestation. Gestational diabetes diagnosed early has also been associated with higher scores on quantitative autism screening measures in children, suggesting associations with broader (subclinical) social and behavioral characteristics.^19^ No studies to date have examined whether risk of NDD other than ASD, such as developmental delay, differs by gestational diabetes diagnosis timing.

Fetal sex is an important potential modifier of prenatal risk factors for NDD given strong evidence for elevated NDD prevalence in males compared with females.^20,21^ Multiple studies have demonstrated sex-specific associations of prenatal cardiometabolic and immune-mediated conditions with child neurodevelopmental outcomes, including obesity or asthma with increased likelihood of NDD in females,^22^reported and gestational diabetes with increased autism-related trait severity among males.^23^ However, most studies have not reported sex-stratified results,^1,24,25,26,27,28,29^ and further investigations of sex-specific associations are warranted.

The objective of this study was to investigate associations between prenatal glucose intolerance and likelihood of child NDD diagnosis and examine the potential modifying roles of gestational timing of exposure onset and child sex. We hypothesized that prenatal glucose intolerance, including IGT, would be associated with higher risk of NDD and that associations would be stronger for gestational diabetes diagnosed early compared with late in gestation and differ by child sex.

## Methods

### Study Population

The study population was selected from children born at Kaiser Permanente Northern California (KPNC) between January 2011 and December 2018, who survived to at least age 2 years, and their mothers with KPNC membership at least 2 years prior to delivery (eFigure 1 in Supplement 1).^22^ Mothers previously consented to provide access to their and their child’s records through participation in the Research Program on Genes, Environment, and Health (RPGEH), a large, racially and ethnically diverse KPNC pregnancy cohort recruited for studies of maternal and child health.^30^ Demographic and clinical information was prospectively recorded in the EHR and extracted on December 31, 2023, when children were aged 5 to 12 years. KPNC is a large integrated health system with a sociodemographic profile representative of the regional population.^31,32^ Study procedures were approved by the KPNC institutional review board and followed Strengthening the Reporting of Observational Studies in Epidemiology (STROBE) reporting guideline.

### Child Neurodevelopmental Outcomes

Children were categorized into 3 mutually exclusive outcome groups: ASD cases, developmental delay cases, and general population controls. ASD diagnoses were based on *Diagnostic and Statistical Manual of Mental Disorders* (Fourth Edition)^33^ or *Diagnostic and Statistical Manual of Mental Disorders* (Fifth Edition)^34^ criteria and recorded in the EHR on at least 1 occasion before the end of follow-up. Children were diagnosed at a KPNC ASD evaluation center by a multidisciplinary team using a standardized protocol, including the Autism Diagnostic Observation Schedule,^35^ or by 1 or more developmental behavioral pediatricians, child psychiatrists, pediatric neurologists, or general pediatricians. Children in the developmental delay group had no diagnosis of ASD and at least 1 of the following diagnoses recorded in the EHR: intellectual disability, cerebral palsy, language delay, motor disorder, global delay, or learning disorder (eTable 1 in Supplement 1). Controls were randomly sampled from children in the RPGEH cohort^30^ who had no diagnoses of ASD or developmental delay recorded in the EHR at an approximately 3:1 ratio to ASD cases and in proportion to the birth-year distribution of cases.

### Prenatal Glucose Intolerance Exposures

Gestational diabetes and IGT were determined from maternal inpatient and outpatient records. All pregnant individuals at KPNC are universally screened for gestational diabetes between approximately 24 to 28 weeks’ gestation,^36^ consistent with US Preventive Services Taskforce (USPSTF) recommendations.^37^ gestational diabetes was defined using data from the KPNC Pregnancy Glucose Tolerance and Gestational Diabetes Registry^36^ based on the following criteria: (1) serum glucose 140 mg/dL or more (to convert to millimoles per liter, multiply by 0.0555) on a 50-g, 1-hour glucose challenge test (GCT), followed by at least 2 serum glucose values meeting or exceeding the Carpenter and Coustan thresholds on the 100-g, 3-hour oral glucose tolerance test (OGTT) during pregnancy (fasting glucose: ≥95 mg/dL; 1 hour glucose: ≥180 mg/dL; 2 hour glucose: ≥155 mg/dL; and 3 hour glucose: ≥140 mg/dL as recommended by American College of Obstetricians and Gynecologists^38^); or (2) 50-g, 1 hour GCT serum glucose of 180 mg/dL or more and a fasting glucose of 95 mg/dL or more performed separate from or during the GCT as recommended by the International Association of Diabetes and Pregnancy Study Groups and American Diabetes Association.^39,40^ Gestational diabetes was further categorized into subgroups based on gestational timing of the diagnosis: early (diagnosis before 24 weeks gestation); standard (diagnosis between 24 and 28 weeks); and late (diagnosis after 28 weeks). IGT was defined by a positive 50-g, 1 hour GCT result of 140 mg/dL or more and no gestational diabetes diagnosis following testing by OGTT. The unexposed group included all individuals who did not meet criteria for gestational diabetes or IGT and were not treated with any antidiabetic medications (eg, glyburide, insulin, or metformin). Individuals with preexisting type 1 or 2 diabetes were excluded.

### Covariates

Study covariates were identified from published evidence of associations with the exposure and/or outcome and associations in our data. These included maternal age at delivery, race and ethnicity, highest education completed, parity, gestational age at first prenatal visit, prepregnancy BMI, and child sex and birth year. Race and ethnicity were self-reported and captured in the medical record by a clinician during initial medical visits; response categories included Asian, Black, Hispanic, White, or other (those who selected a category called other and/or identified with 1 or more race). Prepregnancy BMI was an important potential confounder given its independent associations with both gestational diabetes and ASD. Additional health information used in describing the study sample and secondary analyses included prenatal antidiabetic medication use, preeclampsia, and lifetime history of polycystic ovarian syndrome (PCOS). Missing prepregnancy BMI was imputed by fully conditional specification, a semiparametric imputation method.^41^

### Statistical Analyses

Multivariable logistic regression models were used to estimate crude and adjusted odds ratios (aOR) for ASD vs general population and developmental delay vs general population separately in association with gestational diabetes or IGT in utero, compared with the unexposed referent group (no gestational diabetes and no IGT). Unknown values for categorical sociodemographic and health covariates were included in models as a separate level. We performed stratified analyses by timing of gestational diabetes diagnosis and child sex and tested for group differences using analysis of variance of nested models for timing and interaction terms for sex. In a secondary analysis, we compared associations after stratifying the gestational diabetes exposure group by medication treatment (yes or no), as a proxy for severity. We also performed a sensitivity analysis excluding individuals with PCOS, based on common co-occurrence with gestational diabetes and similarities in recommended treatments.^42,43^ All tests were 2-sided using a significance threshold of *P* < .05 to evaluate main and interaction effects. Analyses occurred between February 2024 and March 2025.

## Results

### Study Population

The study population included 4546 mother-child pairs (683 children with ASD [15.0%]; 2054 with developmental delay [45.2%]; 1809 general population [39.8%]) and was diverse with respect to race and ethnicity (948 Asian [20.9%], 243 Black [5.3%], 1088 Hispanic [23.9%], 2050 White [45.1%], 217 other or unknown [4.8%]), and most mothers (3638 [80.0%]) completed at least some college education (Table 1). The mean (SD) prepregnancy BMI was higher among mothers of children with ASD (28.3 [7.2]) compared with mothers of children with developmental delay (27.0 [6.3]) and mothers of children in the general population (26.9 [6.3]). Higher proportions of mothers in the ASD and developmental delay groups had a history of PCOS (ASD: 33 of 683 [4.8%]; developmental delay: 78 of 2054 [3.8%]; general population: 50 of 1809 [2.8%]). Gestational diabetes prevalence was 10.3% (70 of 683) among mothers in the ASD group, 9.6% (198 of 2054) for developmental delay, and 7.5% (135 of 1809) for general population. Prevalence of IGT was 2.0% (14 of 683) among the ASD group, 1.6% (32 of 2054) among the developmental delay group, and 1.0% (18 of 1809) among the general population group (Table 2). Compared with mothers with neither exposure, those with gestational diabetes or IGT had higher mean prepregnancy BMI and greater proportions had PCOS and reported Asian race (eTable 2 in Supplement 1). All pregnant individuals were screened for gestational diabetes (mean [SD] gestational weeks, 24.7 [5.3] weeks) (eFigure 2 in Supplement 1).

**Table 1.** Summary of Sociodemographic and Health Characteristics of Mother-Child Pairs Included in the Case-Control Study Sample Drawn From Kaiser Permanente Northern California

Characteristic	Overall (n = 4546)	ASD (n = 683)	DD n = 2054)	GP (n = 1809)
Maternal age at birth, mean (SD)	31.2 (5.1)	31.3 (5.2)	31.3 (5.1)	31.0 (5.1)
Maternal race or ethnicity				
Asian	948 (20.9)	153 (22.4)	428 (20.8)	367 (20.3)
Black	243 (5.3)	47 (6.9)	107 (5.2)	89 (4.9)
Hispanic	1088 (23.9)	143 (20.9)	502 (24.4)	443 (24.5)
White	2050 (45.1)	300 (43.9)	930 (45.3)	820 (45.3)
Other or unknown	217 (4.8)	40 (5.9)	87 (4.2)	90 (5.0)
Maternal highest education				
Less than high school	86 (1.9)	9 (1.3)	47 (2.3)	30 (1.7)
High school	562 (12.4)	81 (11.9)	262 (12.8)	219 (12.1)
College	2840 (62.5)	446 (65.3)	1260 (61.3)	1134 (62.7)
Postgraduate	798 (17.6)	113 (16.5)	368 (17.9)	317 (17.5)
Unknown	260 (5.7)	34 (5.0)	117 (5.7)	109 (6.0)
Prepregnancy BMI, mean (SD)	27.1 (6.5)	28.3 (7.2)	27.0 (6.3)	26.9 (6.3)
Preeclampsia	361 (7.9)	71 (10.4)	162 (7.9)	128 (7.1)
History of PCOS	161 (3.5)	33 (4.8)	78 (3.8)	50 (2.8)
Gestational age at first prenatal visit, mean (SD)	7.2 (2.1)	7.2 (2.4)	7.3 (2.2)	7.1 (1.6)
Parity				
0	2038 (44.8)	366 (53.6)	888 (43.2)	784 (43.3)
1	1556 (34.2)	195 (28.6)	720 (35.1)	641 (35.4)
2	615 (13.5)	71 (10.4)	298 (14.5)	246 (13.6)
≥3	238 (5.2)	39 (5.7)	99 (4.8)	100 (5.5)
Missing	99 (2.2)	12 (1.8)	49 (2.4)	38 (2.1)
Child sex				
Male	2697 (59.3)	522 (76.4)	1306 (63.6)	869 (48.0)
Female	1849 (40.7)	161 (23.6)	748 (36.4)	940 (52.0)
Child year of birth				
2011	451 (9.9)	56 (8.2)	219 (10.7)	176 (9.7)
2012	493 (10.8)	66 (9.7)	252 (12.3)	175 (9.7)
2013	875 (19.2)	117 (17.1)	400 (19.5)	358 (19.8)
2014	843 (18.5)	118 (17.3)	399 (19.4)	326 (18.0)
2015	705 (15.5)	109 (16.0)	315 (15.3)	281 (15.5)
2016	523 (11.5)	89 (13.0)	217 (10.6)	217 (12.0)
2017	390 (8.6)	75 (11.0)	145 (7.1)	170 (9.4)
2018	266 (5.9)	53 (7.8)	107 (5.2)	106 (5.9)

Abbreviations: ASD, autism spectrum disorder; BMI, body mass index (calculated as weight in kilograms divided by height in meters squared); DD, developmental delay; GP, general population; PCOS, polycystic ovarian syndrome.

**Table 2.** Results From Crude and Multivariable Logistic Regression Models Estimating Associations Between Prenatal Exposures and Risk of Offspring ASD and DD

Exposure	Mother-child pairs, No. (%)			OR (95% CI)			
	ASD (n = 683)	DD (n = 2054)	GP (n = 1809)	ASD vs GP		DD vs GP	
				Crude	Adjusted^a^	Crude	Adjusted^a^
Unexposed	599 (87.7)	1824 (88.8)	1656 (91.5)	1 [Reference]	1 [Reference]	1 [Reference]	1 [Reference]
GD	70 (10.3)	198 (9.6)	135 (7.5)	1.42 (1.04-1.91)	1.15 (0.83-1.60)	1.32 (1.05-1.67)	1.24 (0.98-1.57)
Early (<24 weeks)	21 (3.1)	51 (2.5)	35 (1.9)	1.64 (0.93-2.81)	1.13 (0.62-2.02)	1.31 (0.85-2.05)	1.16 (0.74-1.84)
Standard (24-28 weeks)	22 (3.2)	77 (3.7)	61 (3.4)	0.98 (0.59-1.59)	0.91 (0.53-1.52)	1.14 (0.81-1.61)	1.09 (0.77-1.55)
Late (>28 weeks)	27 (4.0)	70 (3.4)	39 (2.2)	1.89 (1.14-3.10)	1.52 (0.89-2.57)	1.62 (1.09-2.43)	1.53 (1.03-2.31)
IGT	14 (2.0)	32 (1.6)	18 (1.0)	2.15 (1.04-4.34)	1.87 (0.87-3.95)	1.61 (0.91-2.94)	1.81 (1.00-3.34)

Abbreviations: ASD, autism spectrum disorder; DD, developmental delay; GD, gestational diabetes; GP, general population; IGT, impaired glucose tolerance; OR, odds ratio.

^a^ Models adjusted for child sex and birth year, maternal age at birth, race and ethnicity, education, gestational age at first prenatal care, parity, and prepregnancy body mass index.

### Main Associations

Gestational diabetes was associated with increased odds of ASD and developmental delay in crude models (ASD: OR, 1.42 [95% CI, 1.04-1.91]; developmental delay: OR, 1.32 [95% CI, 1.05-1.67]), but not after adjusting for all covariates (ASD: aOR, 1.13 [95% CI, 0.85-1.50]; developmental delay: aOR, 1.20 [95% CI, 0.98-1.47]) (Table 2 and Figure). Adjusting for sociodemographic variables attenuated associations with both ASD and developmental delay, while adjusting for BMI attenuated associations only with ASD (eTable 3 in Supplement 1). Prenatal exposure to IGT was associated with elevated odds of developmental delay (aOR, 1.81 [95% CI, 1.00-3.34]), but not with ASD (aOR, 1.87 [95% CI, 0.87-3.95]).

**Figure.**
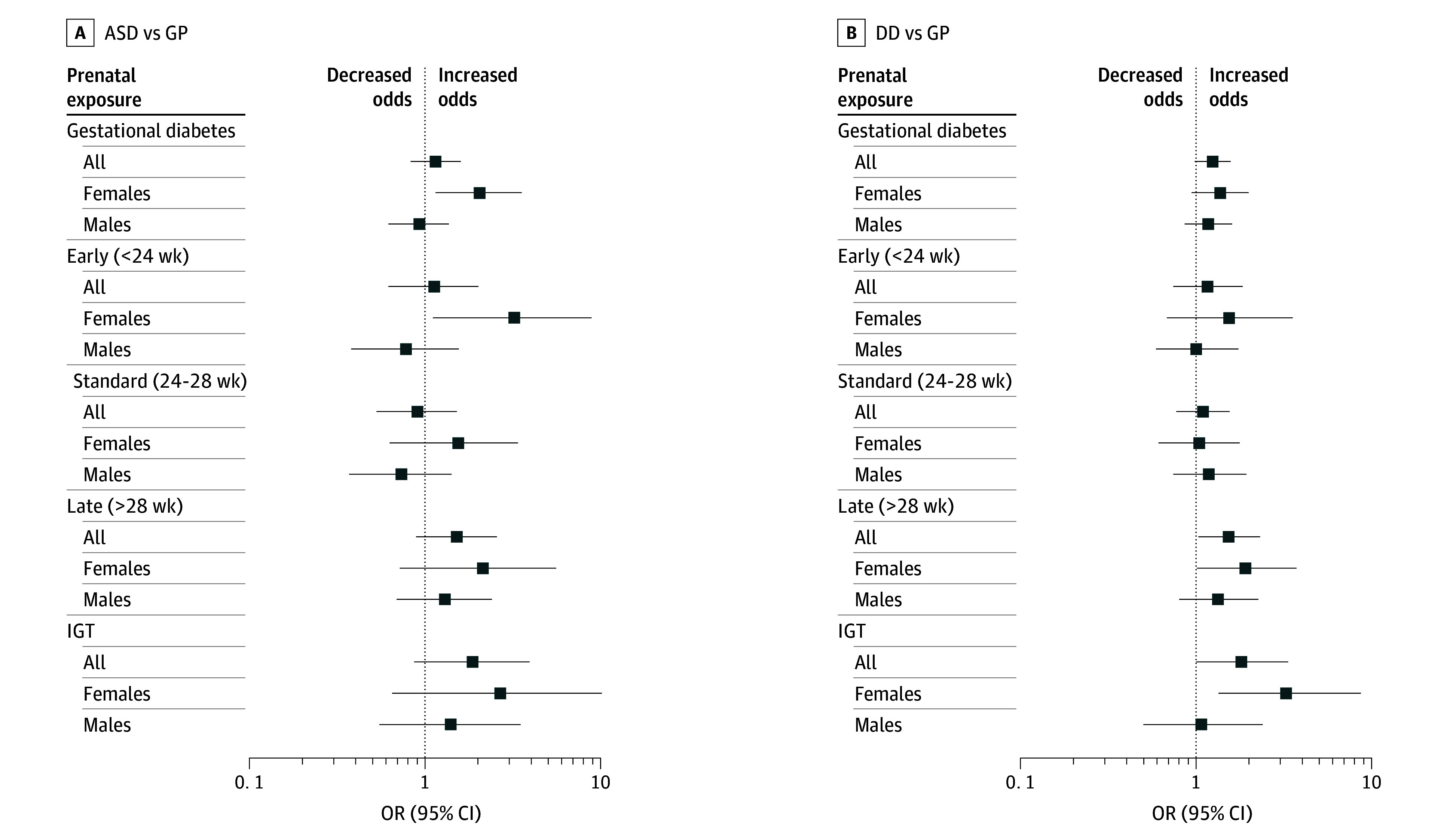
Main and Sex-Stratified Associations of Gestational Diabetes and Subclinical Impaired Glucose Tolerance (IGT) With Odds of Autism Spectrum Disorder (ASD) and Developmental Delay Among 4546 Mother-Child Pairs DD indicates developmental delay; GP, general population; OR, odds ratio.

### Gestational Timing of Gestational Diabetes Diagnosis

After stratifying by gestational timing of diagnosis, associations between gestational diabetes and ASD remained null within each group (less than 24 weeks: aOR, 1.13 [95% CI, 0.62-2.02]; 24 to 28 weeks: aOR, 0.91 [95% CI, 0.53-1.52]; more than 28 weeks: aOR, 1.52 [95% CI, 0.89-2.57]; *P* = .94). Gestational diabetes was associated with increased odds of developmental delay in the late gestational diabetes diagnosis group only (less than 24 weeks: aOR, 1.16 [95% CI, 0.74-1.84]; 24 to 28 weeks: aOR, 1.09 [95% CI, 0.77-1.55]; more than 28 weeks: aOR, 1.53 [95% CI, 1.03-2.31]; *P* for interaction = .40) (Table 2).

### Sex-Stratified Associations

Gestational diabetes was associated with increased odds of ASD among females only (females: aOR, 2.05 [95% CI, 1.15-3.56]; males: aOR, 0.93 [0.62-1.37]; *P* for interaction = .04) whereas associations with developmental delay were similar across sexes (females: aOR, 1.37 [95% CI, 0.94-1.99]; males: aOR, 1.17 [95% CI, 0.86-1.60]; *P* for interaction = .58) (Table 3 and Figure). IGT was not associated with increased odds of ASD in either sex (females: aOR, 2.69 [95% CI, 0.65-10.21]; males: aOR, 1.41 [95% CI, 0.55-3.51]; *P* for interaction = .20) but was associated with increased odds of developmental delay among females only (females: aOR, 3.25 [95% CI, 1.34-8.68]; males: aOR, 1.07 [95% CI, 0.50-2.39]; *P* for interaction = .08).

**Table 3.** Sex-Stratified Associations of GDM and IGT With Odds of ASD and DD Among 4546 Mother-Child Pairs

Exposure	ASD vs GP				*P* value^b^	DD vs GP				*P* value^b^
	Females		Males			Females		Males		
	No.	OR (95% CI)^a^	No.	OR (95% CI)^a^		No.	OR (95% CI)^a^	No.	OR (95% CI)^a^	
GD	21	2.05 (1.15-3.56)	49	0.93 (0.62-1.37)	.04	64	1.37 (0.94-1.99)	134	1.17 (0.86-1.60)	.58
Early (<24 weeks)	7	3.23 (1.11-8.91)	14	0.78 (0.38-1.56)	.02	14	1.54 (0.68-3.55)	37	1.00 (0.59-1.74)	.34
Standard (24-28 weeks)	8	1.55 (0.63-3.39)	14	0.74 (0.37-1.42)	.27	26	1.04 (0.61-1.77)	51	1.18 (0.74-1.93)	.60
Late (>28 weeks)	6	2.14 (0.72-5.60)	21	1.30 (0.69-2.41)	.53	24	1.91 (1.01-3.73)	46	1.33 (0.80-2.26)	.40
IGT	5	2.69 (0.65-10.21)	9	1.41 (0.55-3.51)	.20	15	3.25 (1.34-8.68)	17	1.07 (0.50-2.39)	.08

Abbreviations: ASD, autism spectrum disorder; DD, developmental delay; GD, gestational diabetes; GP, general population; IGT, impaired glucose tolerance; OR, odds ratio.

^a^ Models adjusted for child birth year, maternal age at birth, race and ethnicity, education, gestational age at first prenatal care, parity, and prepregnancy body mass index.

^b^ *P* value for 2-way interaction term between exposure and child sex.

### Gestational Timing of Gestational Diabetes Diagnosis

Additionally stratifying by gestational timing of diagnosis, odds of ASD were significantly increased only among females in the early gestational diabetes group (females: aOR, 3.23 [95% CI, 1.11-8.91]; males: aOR, 0.78 [95% CI, 0.38-1.56]; *P* for interaction = .02) and not in the standard- or late-gestation diagnosis groups for either sex (24 to 28 weeks: aOR for females, 1.55 [95% CI, 0.63-3.39]; aOR for males, 0.74 [95% CI, 0.37-1.42]; *P* for interaction = .27; >28 weeks: aOR for females = 2.14 [95% CI, 0.72-5.60]; aOR for males, 1.30 [95% CI, 0.69-2.41]; *P* for interaction = .53). Odds of developmental delay were also significantly elevated among females and only in the late gestational diabetes group (before 24 weeks gestation: aOR for females, 1.54 [95% CI, 0.68-3.55]; aOR for males, 1.00 [95% CI, 0.59-1.74]; *P* for interaction = .34; 24-28 weeks gestation: aOR for females, 1.04 [95% CI, 0.61-1.77]; aOR for males, 1.18 [95% CI, 0.74-1.93]; *P* for interaction = .40; after 28 weeks gestation: aOR for females, 1.91 [95% CI, 1.01-3.73]; aOR for males, 1.33 [95% CI, 0.80-2.26]; *P* for interaction = .40) (Table 3).

### Stratification by Antidiabetic Medication Treatment

Proportions of mothers with gestational diabetes who were treated with antidiabetic medication were similar across outcome groups (ASD: 35 of 70 [50.0%]; developmental delay: 98 of 198 [49.5%]; general population: 66 of 135 [48.9%]). Compared with untreated, those treated with medication had higher prepregnancy BMI and greater proportions with PCOS and early gestational diabetes diagnosis (before 24 weeks gestation) (eTable 4 in Supplement 1). There were no differences across treated and untreated groups in the odds of ASD or developmental delay associated with prenatal glucose intolerance from fully adjusted models (eTable 5 in Supplement 1).

### Exclusion of Individuals With PCOS

Of the 4546 pregnant individuals, 161 (3.5%) had a diagnosis of PCOS. Of those, 42 (24.6%) had gestational diabetes and 2 (1.2%) had IGT. Results were consistent with those from main analyses after excluding individuals with PCOS (eTable 6 in Supplement 1). For example, there were no associations of gestational diabetes with ASD (aOR, 1.14 [95% CI, 0.80-1.59]) or developmental delay (aOR, 1.18 [95% CI, 0.92-1.51]) and IGT remained associated with increased odds of developmental delay (aOR, 1.86 [95% CI, 1.03-3.51]).

## Discussion

Prenatal glucose intolerance was associated with increased risk of child NDD in a manner that is both timing- and sex-specific for gestational diabetes and sex-specific for IGT. Gestational diabetes diagnosed earlier in pregnancy and IGT were associated with increased risk of NDD among females only, suggesting underlying mechanisms may be sexually dimorphic.

### Comparison With Past Research

Past studies of associations between gestational diabetes and ASD have been mixed, with some reporting no association,^22,44,45,46^ and others increased risk.^1,47,48^ Potential reasons for these discrepancies include differences in sample size, child sex ratio, gestational diabetes exposure ascertainment (eg, diagnostic criteria, clinical vs self-reported), and covariate adjustment. Adjustment for prepregnancy BMI, a known risk factor for both gestational diabetes^49^ and child NDD,^50^ attenuated risk of ASD in our study but was not accounted for in 2 large studies reporting increased risk of ASD^1,47^ nor in a previous analysis of a subset of the current study sample reporting increased risk of developmental delay.^22^ Future studies should carefully consider the role of prepregnancy BMI in associations with between gestational diabetes and NDDs. A recent meta-analysis of more than 200 observational studies also highlighted the importance of consistent confounder adjustment, demonstrating greater attenuation of NDD risk associated with gestational diabetes in studies adjusting for 3 or more confounders compared with 1 or fewer confounders.^5^ No past studies to our knowledge excluded an IGT or similar exposure subgroup from the reference group, which could have conflated milder hyperglycemia with normoglycemia and attenuated risk estimates.

### Gestational Timing of Gestational Diabetes Diagnosis

Previous studies^1^ suggested early diagnosis (before 26 weeks gestation) could be an indicator of earlier onset or severe gestational diabetes, and that associated increased risk of ASD may be due to prolonged exposure to hyperglycemia overlapping critical developmental periods. Maternal metabolic dysregulation and neuroinflammation in early pregnancy can adversely impact fetal development and first- and second-trimester specific risks with NDD have been reported for other prenatal conditions including infection, asthma, and allergy.^51,52,53^ In contrast, we found late diagnosis (after 28 weeks’ gestation) but not early diagnosis (before 24 weeks gestation) of gestational diabetes was associated with increased odds of developmental delay. This could reflect prolonged exposure to hyperglycemia prior to treatment following gestational diabetes diagnosis. Relatively small samples of cases in the exposed groups, particularly for ASD, limited precision of these findings and further research is warranted.

### Prenatal Impaired Glucose Tolerance

Prenatal IGT identified by laboratory results as a separate exposure from gestational diabetes was associated with significantly increased risk of developmental delay among females only. Despite not receiving a gestational diabetes diagnosis, pregnant individuals with subclinical indicators of hyperglycemia may still experience potentially harmful metabolic dysregulation particularly because this condition is generally untreated. These individuals and their children may therefore benefit from lifestyle and treatment recommendations similar to those for gestational diabetes^17^ to improve maternal health and inform treatment of high likelihood child subgroups. Larger studies are needed to test this hypothesis.

### Sex Differences

Risks of NDD were consistently higher among females than males, particularly for ASD and among those exposed to gestational diabetes early in gestation. A growing body of evidence suggests fetal sex is an important modifier of gestational susceptibility to inflammation by influencing placental and maternal inflammatory responses^54,55,56,57^ as well as the risk of gestational diabetes.^58^ Similar roles of fetal sex have been demonstrated for other maternal conditions that may induce maternal inflammation through metabolic and/or immune dysregulation, including preterm delivery,^59^ preeclampsia,^60^ and asthma.^61,62^ It is therefore plausible that the fetal-maternal unit responds to gestational diabetes and IGT in a fetal sex-dependent manner.

Our sex-specific findings are consistent with a previous analysis of a subset of our case-control sample of 2569 mother-child pairs, which reported elevated NDD risk among females exposed to inflammatory conditions co-occurring with metabolic dysfunction, including allergy, asthma, obesity, and hypertension.^22^ Other studies have reported little or no evidence for child sex modification of NDD risk associated with gestational diabetes or other cardiometabolic factors,^63,64^ and further research is warranted, especially considering inconsistencies across studies in exposure and NDD outcome ascertainment.^5^

### Strengths and Limitations

Prenatal glucose intolerance was defined from laboratory test results, which enabled subgroup analyses by gestational timing of diagnosis and novel investigation of IGT as a separate exposure. The use of 3 gestational timing groups, compared with prior studies that used a single 26-week cut point,^1,2,19^ may also better reflect critical periods of exposure relevant to neurodevelopment. We were also able to rule out potential confounding by multiple commonly co-occurring health conditions, including obesity and PCOS, lending further support to our findings.

This study has limitations. Some proportion of those diagnosed with gestational diabetes late may actually have had earlier gestational diabetes onset, in which case our estimates of risk associated with early diagnosis would be biased toward the null. Individuals at high risk for gestational diabetes may have been systematically screened earlier and therefore more likely to be diagnosed early, and we accounted for this as best as possible by controlling for known gestational diabetes risk factors and gestational age at first prenatal care visit. Consistent with the rationale presented by Rothman^65^ and Savitz,^66^ we did not account for multiple comparisons because primary hypotheses were prespecified and based on results of prior studies. We cannot rule out that some study findings may be due to chance, and larger follow-up studies are warranted, especially considering the small sample sizes in stratified analyses. We were unable to disaggregate the developmental delay group into specific conditions (eg, cerebral palsy, motor delay) or examine child ADHD, which has been associated with gestational diabetes but cannot be reliably diagnosed until later in childhood.^3,67^ Finally, the KPNC population is representative of northern California and may not be generalizable to areas in the US with different sociodemographic compositions.

## Conclusions

In case-control data ascertained from an integrated health system, we found associations between prenatal glucose intolerance and NDDs differed by gestational timing and child sex. Our findings suggest that pregnant individuals with elevated screening glucose levels who do not receive a diagnosis of gestational diabetes could benefit from treatment recommendations and further research is warranted.

## Notes

Supplement 1. **eTable 1.** Definitions of child neurodevelopmental disorders **eTable 2.** Summary of sociodemographic and health characteristics **eTable 3.** Results from crude and adjusted models showing attenuation of associations between prenatal glucose intolerance and child NDDs **eTable 4.** Study population characteristics among those with GDM, overall and stratified by treatment with antidiabetic medication **eTable 5. **Associations of GDM with ASD and DD from secondary analyses, after stratifying the GDM exposure group by treatment with any antidiabetic medication during pregnancy **eTable 6. **Associations between prenatal exposures and odds of ASD and DD before after excluding 161 individuals with a history of PCOS **eFigure 1. **Flowchart of study sample ascertainment from Kaiser Permanente Northern California **eFigure 2. **Distribution of gestational age at gestational diabetes screening for 4546 mothers in the study sample
eReferences

Supplement 2.
Data Sharing Statement
