# Supplementary material for: Prenatal Glucose Intolerance and Child Neurodevelopmental Disorders
Source: JAMA Netw Open. 2025 Nov 5;8(11):e2541657. doi: 10.1001/jamanetworkopen.2025.41657 (PMC12590297; doi:10.1001/jamanetworkopen.2025.41657)
Supplement: Supplement 1. — eTable 1. Definitions of child neurodevelopmental disorders eTable 2. Summary of sociodemographic and health characteristics eTable 3. Results from crude and adjusted models showing attenuation of associations between prenatal glucose intolerance and child NDDs eTable 4. Study population characteristics among those with GDM, overall and stratified by treatment with antidiabetic medication eTable 5. Associations of GDM with ASD and DD from secondary analyses, after stratifying the GDM exposure group by treatment with any antidiabetic medication during pregnancy eTable 6. Associations between prenatal exposures and odds of ASD and DD before after excluding 161 individuals with a history of PCOS eFigure 1. Flowchart of study sample ascertainment from Kaiser Permanente Northern California eFigure 2. Distribution of gestational age at gestational diabetes screening for 4546 mothers in the study sample eReferences [file jamanetwopen-e2541657-s001.pdf]

Supplemental Online Content

Grosvenor LP, Gunderson EP, Qian Y, et al. Prenatal glucose intolerance and child neurodevelopmental disorders. *JAMA Netw Open*. 2025;8(11):e2541657. doi:10.1001/jamanetworkopen.2025.41657

eTable 1. Definitions of child neurodevelopmental disorders
eTable 2. Summary of sociodemographic and health characteristics
eTable 3. Results from crude and adjusted models showing attenuation of associations between prenatal glucose intolerance and child NDDs
eTable 4. Study population characteristics among those with GDM, overall and stratified by treatment with antidiabetic medication
eTable 5. Associations of GDM with ASD and DD from secondary analyses, after stratifying the GDM exposure group by treatment with any antidiabetic medication during pregnancy
eTable 6. Associations between prenatal exposures and odds of ASD and DD before after excluding 161 individuals with a history of PCOS
eFigure 1. Flowchart of study sample ascertainment from Kaiser Permanente Northern California
eFigure 2. Distribution of gestational age at gestational diabetes screening for 4546 mothers in the study sample
eReferences

This supplemental material has been provided by the authors to give readers additional information about their work.

eTable 1. Definitions of child neurodevelopmental disorders.

**Condition**	**ICD Codes**	
Autism Spectrum Disorder (ASD)	ICD-9	299.0, 299.8, 299.9
	ICD-10	F84.0, F84.5, F84.8, F84.9
Developmental Delay (DD)		Any ID, CP, Language Delay, Motor Disorder, Global Delay, or Learning Disorder diagnosis as defined below
Intellectual Disability (ID)	ICD-9	317-319
	ICD-10	F70-F73, F78, F79
Cerebral Palsy (CP)	ICD-9	333.71, 342.1, 342.8, 342.9, 343.0-343.9, 344.0, 344.1, 344.30, 344.31, 344.32, 344.5
	ICD-10	G04.1, G80.0 - G80.4, G80.8, G80.9, G81.1, G81.9, G82.20-G82.22, G82.5, G83.10 - G83.14, G83.30 - G83.34, R25.8
Language Delay	ICD-9	315.3
	ICD-10	F80.0-F80.2, F80.4, F80.82, F80.89, F80.9, H93.25
Motor Disorder	ICD-9	315.4
	ICD-10	F82
Global Delay	ICD-9	315.5, 315.8, 315.9
	ICD-10	F81.9, F88, F89
Learning Disorder	ICD-9	315.0, 315.1, 315.2
	ICD-10	F81.0, F81.2, F81.81, F81.89, R48.0

eTable 2. Summary of sociodemographic and health characteristics of the mother-child pairs included in the study sample (N=4,546) stratified by prenatal exposure to gestational diabetes or impaired glucose tolerance.

**Characteristic**	**Overall (N=4,546)**	**GDM (N=403)**	**IGT (N=64)**	**Unexposed (N=4,079)**
Maternal Age at Birth (mean (SD))	31.2 (5.1)	33.1 (5.0)	31.6 (5.3)	31.0 (5.1)
Maternal Race/Ethnicity, N (%)				
Asian	948 (20.9)	144 (35.7)	22 (34.4)	782 (19.2)
Black	243 (5.3)	14 (3.5)	1 (1.6)	228 (5.6)
Hispanic	1088 (23.9)	101 (25.1)	14 (21.9)	973 (23.9)
Unknown	217 (4.8)	19 (4.7)	6 (9.4)	192 (4.7)
White	2050 (45.1)	125 (31.0)	21 (32.8)	1904 (46.7)
Maternal Highest Education, N (%)				
Under High School	86 (1.9)	11 (2.7)	1 (1.6)	74 (1.8)
High School	562 (12.4)	44 (10.9)	10 (15.6)	508 (12.5)
College	2840 (62.5)	251 (62.3)	46 (71.9)	2543 (62.3)
Post-Graduate	798 (17.6)	71 (17.6)	4 (6.2)	723 (17.7)
Unknown	260 (5.7)	26 (6.5)	3 (4.7)	231 (5.7)
Pre-pregnancy BMI (mean (SD))	27.1 (6.5)	29.9 (7.0)	29.5 (6.8)	26.8 (6.3)
Preeclampsia, N (%)	361 (7.9)	54 (13.4)	8 (12.5)	299 (7.3)
History of PCOS, N (%)	161 (3.5)	33 (8.2)	2 (3.1)	126 (3.1)
Gestational age at first prenatal visit (mean (SD))	7.2 (2.0)	7.3 (2.1)	7.0 (1.8)	7.2 (2.0)
Parity, N (%)				
0	2038 (44.8)	160 (39.7)	22 (34.4)	1856 (45.5)
1	1556 (34.2)	127 (31.5)	27 (42.2)	1402 (34.4)
2	615 (13.5)	71 (17.6)	10 (15.6)	534 (13.1)
3+	238 (5.2)	35 (8.7)	3 (4.7)	200 (4.9)
Missing	99 (2.2)	10 (2.5)	2 (3.1)	87 (2.1)
Child Sex Male, N (%)	2697 (59.3)	258 (64.0)	37 (57.8)	2402 (58.9)
Child Year of Birth, N (%)				
2011	451 (9.9)	31 (7.7)	3 (4.7)	417 (10.2)
2012	493 (10.8)	40 (9.9)	2 (3.1)	451 (11.1)
2013	875 (19.2)	74 (18.4)	6 (9.4)	795 (19.5)
2014	843 (18.5)	85 (21.1)	6 (9.4)	752 (18.4)
2015	705 (15.5)	70 (17.4)	6 (9.4)	629 (15.4)
2016	523 (11.5)	52 (12.9)	14 (21.9)	457 (11.2)
2017	390 (8.6)	25 (6.2)	14 (21.9)	351 (8.6)
2018	266 (5.9)	26 (6.5)	13 (20.3)	227 (5.6)

GDM=gestation diabetes; IGT=impaired glucose tolerance; ASD=autism spectrum disorder; DD=developmental delay; GP=general population control

eTable 3. Results from crude and adjusted models showing attenuation of associations between prenatal glucose intolerance and child NDDs following adjustment for sociodemographic and health covariates.

	ASD versus GP, OR (95% CI)			DD versus GP, OR (95% CI)		
**Exposure**	**Crude**	**Model 1[a]**	**Model 2[b]**	**Crude**	**Model 1[a]**	**Model 2[b]**
GDM	1.42 (1.04-1.91)	1.27 (0.91-1.75)	1.15 (0.83-1.60)	1.32 (1.05-1.67)	1.25 (0.99-1.58)	1.24 (0.98-1.57)
Early (<24 weeks)	1.64 (0.93-2.81)	1.34 (0.75-2.37)	1.13 (0.62-2.02)	1.31 (0.85-2.05)	1.16 (0.74-1.82)	1.16 (0.74-1.84)
Standard (24-30 weeks)	0.98 (0.59-1.59)	1.04 (0.61-1.72)	0.91 (0.53-1.52)	1.14 (0.81-1.61)	1.11 (0.78-1.58)	1.09 (0.77-1.55)
Late (>28 weeks)	1.89 (1.14-3.10)	1.63 (0.96-2.74)	1.52 (0.89-2.57)	1.62 (1.09-2.43)	1.55 (1.04-2.34)	1.53 (1.03-2.31)
IGT	2.15 (1.04-4.34)	2.01 (0.93-4.25)	1.87 (0.87-3.95)	1.61 (0.91-2.94)	1.79 (1.00-3.32)	1.81 (1.00-3.34)

GDM=gestation diabetes; IGT=impaired glucose tolerance; ASD=autism spectrum disorder; DD=developmental delay; GP=general population control.

[a]Adjusted for child sex and birth year, maternal age at birth, race/ethnicity, education, gestational age at first prenatal care, and parity

[b]Adjusted for Model 1 covariates plus pre-pregnancy BMI

eTable 4. Study population characteristics among those with GDM, overall and stratified by treatment with antidiabetic medication.

Characteristic	Overall (N=403)	Medication (N=199)	No Medication (N=204)
Maternal Age at Birth (mean (SD))	33.08 (4.95)	33.35 (4.99)	32.82 (4.90)
Maternal Race/Ethnicity, N (%)			
Asian	144 (35.7)	73 (36.7)	71 (34.8)
Black	14 (3.5)	10 (5.0)	4 (2.0)
Hispanic	101 (25.1)	49 (24.6)	52 (25.5)
Unknown	19 (4.7)	10 (5.0)	9 (4.4)
White	125 (31.0)	57 (28.6)	68 (33.3)
Maternal Highest Education, N (%)			
Under High School	11 (2.7)	2 (1.0)	9 (4.4)
High School	44 (10.9)	23 (11.6)	21 (10.3)
College	251 (62.3)	131 (65.8)	120 (58.8)
Post-Graduate	71 (17.6)	32 (16.1)	39 (19.1)
Unknown	26 (6.5)	11 (5.5)	15 (7.4)
Pre-pregnancy BMI (mean (SD))	29.93 (7.01)	31.51 (7.04)	28.38 (6.65)
Preeclampsia, N (%)	54 (13.4)	27 (13.6)	27 (13.2)
History of PCOS, N (%)	33 (8.2)	21 (10.6)	12 (5.9)
Child Sex Male, N (%)	258 (64.0)	126 (63.3)	132 (64.7)
Child Year of Birth, N (%)			
2011	31 (7.7)	10 (5.0)	21 (10.3)
2012	40 (9.9)	17 (8.5)	23 (11.3)
2013	74 (18.4)	37 (18.6)	37 (18.1)
2014	85 (21.1)	44 (22.1)	41 (20.1)
2015	70 (17.4)	35 (17.6)	35 (17.2)
2016	52 (12.9)	29 (14.6)	23 (11.3)
2017	25 (6.2)	17 (8.5)	8 (3.9)
2018	26 (6.5)	10 (5.0)	16 (7.8)
Timing of GDM diagnosis, N (%)			
Early, <24 weeks	107 (26.6)	76 (38.2)	31 (15.2)
Standard, 24-28 weeks	160 (39.7)	75 (37.7)	100 (49.0)
Late >28 weeks	136 (33.7)	48 (24.1)	69 (33.8)
NDD Diagnosis, N (%)			
ASD	70 (17.4)	35 (17.6)	35 (17.2)
DD	198 (49.1)	98 (49.2)	100 (49.0)
POP	135 (33.5)	66 (33.2)	69 (33.8)
Parity, N (%)			
0	160 (39.7)	80 (40.2)	80 (39.2)
1	127 (31.5)	62 (31.2)	65 (31.9)
2	71 (17.6)	37 (18.6)	34 (16.7)
3+	35 (8.7)	17 (8.5)	18 (8.8)
Missing	10 (2.5)	3 (1.5)	7 (3.4)

eTable 5. Associations of GDM with ASD and DD from secondary analyses, after stratifying the GDM exposure group by treatment with any antidiabetic medication during pregnancy.

		Medication		No medication	
**Outcome**	**Exposure**	**Adjusted OR[a] (95% CI)**	**P value**	**Adjusted OR[a] (95% CI)**	**P value**
**ASD**	GDM	1.13 (0.71-1.77)	.61	1.25 (0.79-1.93)	.33
	Early (<24 weeks)	1.14 (0.55-2.29)	.72	1.20 (0.39-3.33)	.74
	Standard (24-28 weeks)	0.95 (0.43-1.96)	.95	0.94 (0.44-1.88)	.86
	Late (>28 weeks)	1.43 (0.56-3.55)	.44	1.61 (0.84-3.05)	.15
**DD**	GDM	1.31 (0.94-1.83)	.12	1.21 (0.88-1.67)	.26
	Early (<24 weeks)	1.29 (0.76-2.22)	.36	0.92 (0.41-2.11)	.84
	Standard (24-28 weeks)	1.12 (0.67-1.88)	.66	1.10 (0.68-1.77)	.70
	Late (>28 weeks)	1.73 (0.89-3.52)	.12	1.46 (0.90-2.44	.13

[a]Models adjusted for child sex and birth year, maternal age at birth, race/ethnicity, education, gestational age at first prenatal care, parity, and pre-pregnancy BMI

eTable 6. Associations between prenatal exposures and odds of ASD and DD before after excluding 161 individuals with a history of PCOS from the analytic sample (N=4,385).

Exposure	ASD (N=650) N (%)	DD (N=1,976) N (%)	GP (N=1,759) N (%)	ASD versus GP OR[a] (95% CI)	DD versus GP OR[a] (95% CI)
Unexposed	573 (88.2)	1766 (89.4)	1614 (91.7)	REF	REF
GDM	63 (9.7)	179 (9.1)	128 (7.3)	1.14 (0.80-1.59)	1.18 (0.92-1.51)
Early (<24 weeks)	18 (3.1)	42 (2.1)	32 (1.8)	1.15 (0.60-2.13)	1.03 (0.64-1.68)
Standard (24-28 weeks)	21 (3.7)	75 (3.8)	58 (3.3)	0.96 (0.55-1.62)	1.13 (0.79-1.63)
Late (>28 weeks)	24 (4.2)	63 (3.2)	38 (2.2)	1.46 (0.84-2.51)	1.41 (0.93-2.14)
IGT	14 (2.2)	31 (1.6)	17 (1.0)	2.05 (0.95-4.39)	1.86 (1.02-3.51)

ASD=autism spectrum disorder; DD=developmental delay; GDM=gestation diabetes; IGT=impaired glucose tolerance; OR=odds ratio.
[a]Models adjusted for child sex and birth year, maternal age at birth, race/ethnicity, education, gestational age at first prenatal care, parity, and pre-pregnancy BMI

eFigure 1. Flowchart of study sample ascertainment from Kaiser Permanente Northern California.

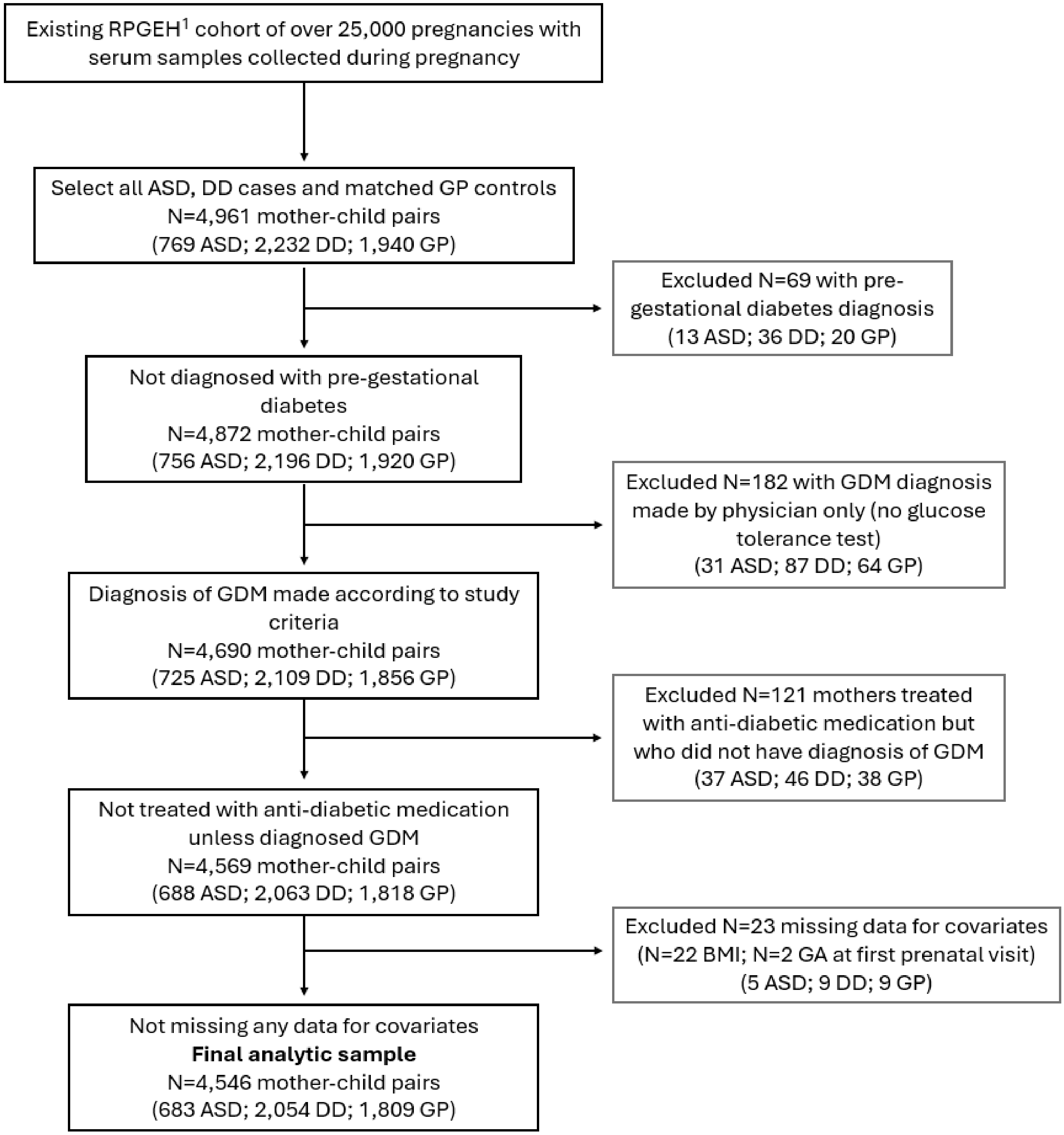

RPGEH=Research Program on Genes, Environment, and Health; ASD=autism spectrum disorder; DD=developmental delay; GP=general population control; GDM=gestational diabetes; BMI=body mass index; GA=gestational age (weeks)

eFigure 2. Distribution of gestational age at gestational diabetes screening for 4,546 mothers in the study sample (dashed line: mean=24.7 weeks; standard deviation=5.3).

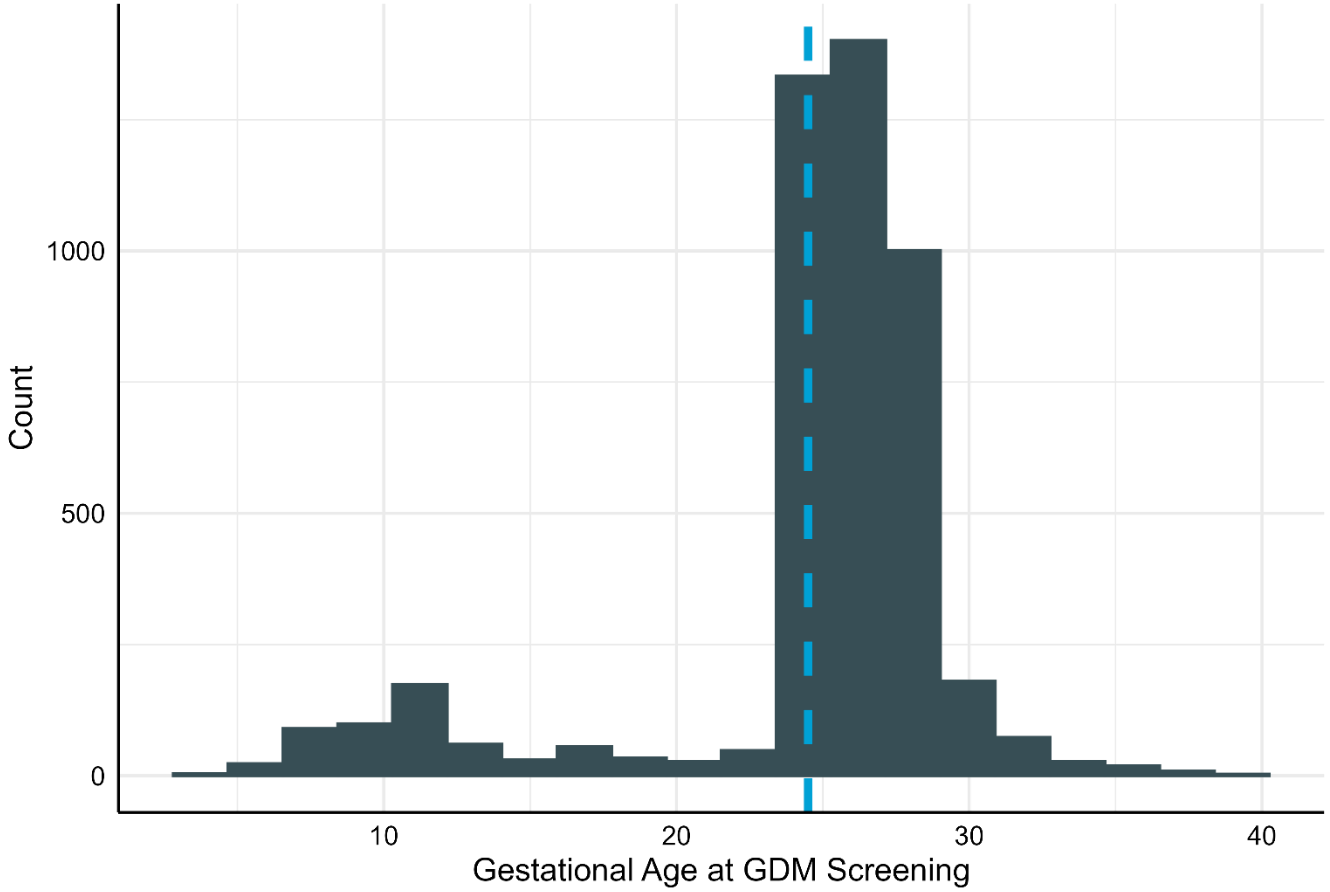

eReferences

1. Hedderson MM, Ferrara A, Avalos LA, et al. The Kaiser Permanente Northern California research program on genes, environment, and health (RPGEH) pregnancy cohort: study design, methodology and baseline characteristics. *BMC Pregnancy Childbirth*. Nov 29 2016;16(1):381. doi:10.1186/s12884-016-1150-2
