# Supplementary material for: Prenatal Glucose Intolerance and Child Neurodevelopmental Disorders
Source: JAMA Netw Open. 2025 Nov 5;8(11):e2541657. doi: 10.1001/jamanetworkopen.2025.41657 (PMC12590297; doi:10.1001/jamanetworkopen.2025.41657)
Supplement: Supplement 2. — Data Sharing Statement [file jamanetwopen-e2541657-s002.pdf]

Data Sharing Statement

Grosvenor. Prenatal Glucose Intolerance and Child Neurodevelopmental Disorders. *JAMA Netw Open*. Published November 05, 2025. doi:10.1001/jamanetworkopen.2025.41657

Data
Data available: No

Additional Information
Explanation for why data not available: The datasets generated during and/or analyzed during the current study are not publicly available due to potentially identifiable information (e.g., dates of diagnoses) and KPNC privacy regulations. They are available from the corresponding author upon reasonable request, and contingent on appropriate human subjects' approval and data use agreements.
